# Biosynthesis of the Diterpenoid Lycosantalonol via Nerylneryl Diphosphate in *Solanum lycopersicum*

**DOI:** 10.1371/journal.pone.0119302

**Published:** 2015-03-18

**Authors:** Yuki Matsuba, Jiachen Zi, A. Daniel Jones, Reuben J. Peters, Eran Pichersky

**Affiliations:** 1 Department of Molecular, Cellular and Developmental Biology, University of Michigan, Ann Arbor, Michigan, United States of America; 2 Department of Biochemistry, Biophysics & Molecular Biology, Iowa State University, Ames, Iowa, United States of America; 3 Department of Biochemistry, Michigan State University, East Lansing, Michigan, United States of America; University of Copenhagen, DENMARK

## Abstract

We recently reported that three genes involved in the biosynthesis of monoterpenes in trichomes, a *cis*-prenyltransferase named neryl diphosphate synthase 1 (*NDPS1*) and two terpene synthases (*TPS19* and *TPS20*), are present in close proximity to each other at the tip of chromosome 8 in the genome of the cultivated tomato (*Solanum lycopersicum*). This terpene gene “cluster” also contains a second *cis*-prenyltransferase gene (*CPT2*), three other TPS genes, including *TPS21*, and the cytochrome P450-oxidoreductase gene *CYP71BN1*. *CPT2* encodes a neryneryl diphosphate synthase. Co-expression in *E*. *coli* of *CPT2* and *TPS21* led to the formation of the diterpene lycosantalene, and co-expression in *E*. *coli* of *CPT2*, *TPS21* and CYP71BN1 led to the formation of lycosantalonol, an oxidation product of lycosantalene. Here we show that maximal expression of all three genes occurs in the petiolule part of the leaf, but little expression of these genes occurs in the trichomes present on the petiolules. While lycosantalene or lycosantalonol cannot be detected in the petiolules of wild-type plants (or anywhere else in the plant), lycosantalene and lycosantalonol are detected in petiolules of transgenic tomato plants expressing *CPT2* under the control of the 35S CaMV promoter. These results suggest that lycosantalene and lycosantalonol are produced in the petiolules and perhaps in other tissues of wild-type plants, but that low rate of synthesis, controlled by the rate-limiting enzyme CPT2, results in product levels that are too low for detection under our current methodology. It is also possible that these compounds are further modified in the plant. The involvement of *CPT2*, *TPS21* and *CYP71BN1* in a diterpenoid biosynthetic pathway outside the trichomes, together with the involvement of other genes in the cluster in the synthesis of monoterpenes in trichomes, indicates that this cluster is further evolving into “sub-clusters” with unique biochemical, and likely physiological, roles.

## Introduction

The metabolic pathways that are shared by practically all plant species are generally termed primary metabolism, while new biochemical pathways that have evolved in various plant lineages in response to selection exerted by local biotic and abiotic factors have been dubbed “specialized” metabolism [1]. Terpenoids constitute a large class of plant metabolites, and while some terpenoids belong to primary metabolism, such as the sterols, carotenoids, and gibberellins, the majority of them have limited distribution in specific plant lineages and are thus part of specialized metabolism.

In particular, thousands of monoterpene, sesquiterpene and diterpene compounds have been identified in various plants serving diverse functions such as floral scents, defense compounds throughout the plants, and signal molecules [2–6]. The diversity is achieved mostly by divergence in the terpene synthase (TPS) gene family among plant species. This family, which includes anywhere from a score to over a hundred genes in any given plant genome, encodes enzymes that use prenyldiphosphates as precursors to fashion a basic hydrocarbon backbone, which can then be further modified by hydroxylation, glycosylation, acylation, peroxidation, cleavage, and other reactions [7].

Most TPSs use *trans*-prenyl diphosphates as their precursor—geranyl diphosphate (GPP) for monoterpene synthases, *E*,*E*-farnesyl diphosphate (*ee*FPP) for sesquiterpene synthases, and *E*,*E*,*E*-geranylgeranyl diphosphate (GGPP) for diterpene synthases. However, it was recently discovered that some TPSs of plants in the Solanaceae family use *cis*-prenyl diphosphates as substrates—neryl diphosphate (NPP) for monoterpenes, *Z*,*Z*-farnesyl diphosphate (*zz*FPP) for sesquiterpene synthases, and neryneryl diphosphate (NNPP) for diterpene synthases [8–11]. Thus, a change in substrates also adds to the diversity of terpenes found in nature.

**Fig 1:**
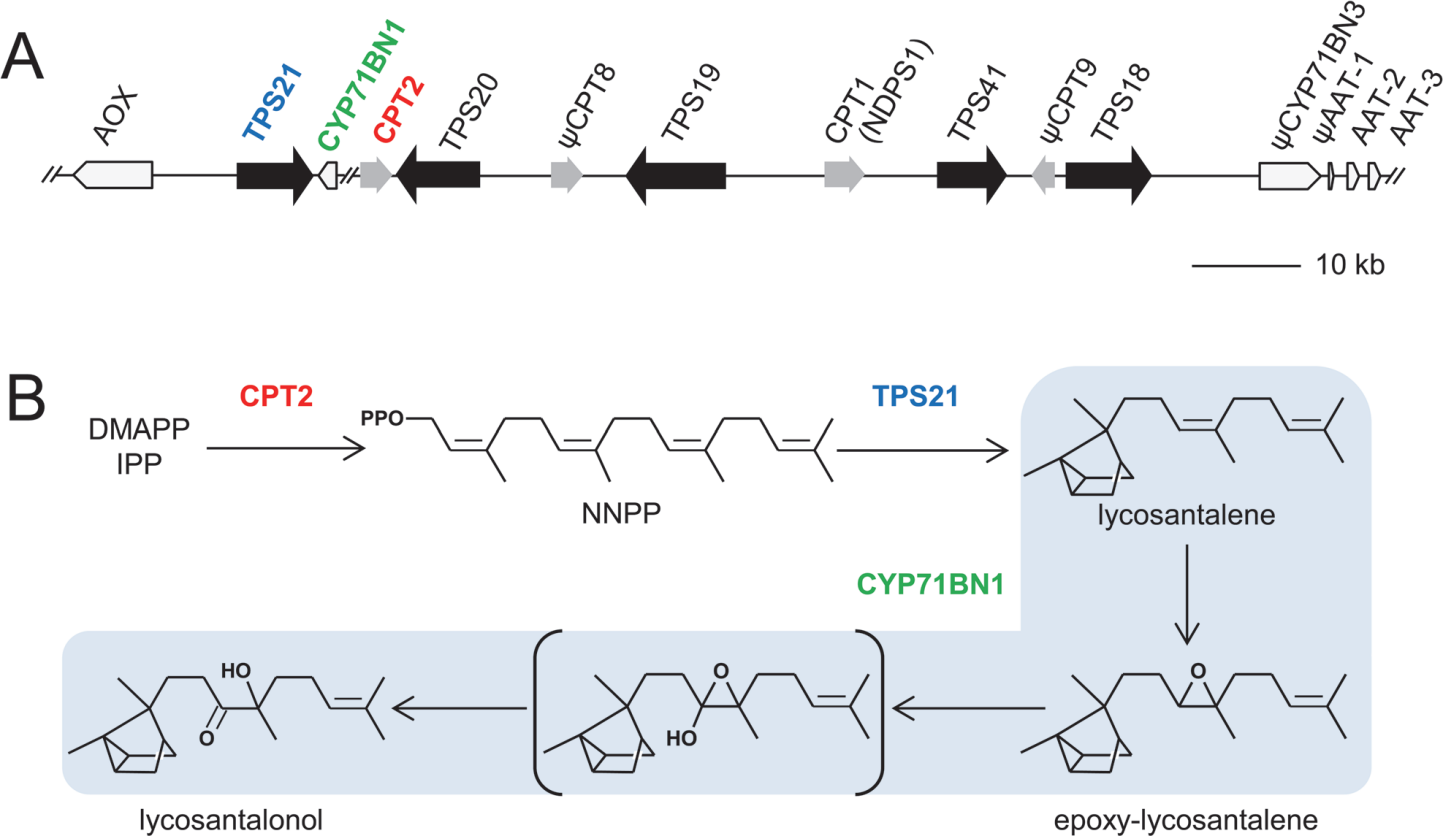
Lycosantalonol biosynthesis in *Solanum lycopersicum*. (**A**) The terpene gene cluster on the tip of chromosome 8. (**B**) The biosynthetic pathway to lycosantanolol. AOX, alcohol oxidase; TPS, terpene synthase; CPT, *cis*-prenyl transferase; NDPS1, neryl diphosphate synthase 1; CYP, cytochrome P450; AAT, alcohol acyltransferase; DMAPP, dimethylallyl diphosphate; IPP, isopentenyl diphosphate; NNPP, nerylneryl diphosphate. Genes that are not functional because of deletions or insertions are shown with a “ψ” symbol.

A detailed analysis of the TPS gene family in *Solanum lycopersicum* (cultivated tomato) identified a cluster of genes on chromosome 8 (Fig. 1A, [11]) that include five TPS genes as well as two functional *cis*-prenyltransferases (= *cis*-prenyl diphosphate synthases) and one functional cytochrome P450 oxidoreductase gene, previously designated as *CYP71D51* [12] but renamed here as *CYP71BN1* according to the numbering assignment of the Cytochrome P450 homepage (http://drnelson.uthsc.edu/CytochromeP450.html). Two of the TPS genes, *TPS19* and *TPS20*, were shown to encode monoterpene synthases that use NPP, the product of *CPT1* (= neryl diphosphate synthase 1, or *NDPS1*). *TPS19*, *TPS20* and *NDPS1* are highly expressed in trichomes, which synthesize and accumulate the resulting monoterpenes [8].

However, the other TPS genes in this cluster were not specifically or even highly expressed in trichomes [11]. Two of these TPSs, *TPS18* and *TPS41*, were shown to be closely related to a pair of *Nicotiana tobacum* (tobacco) genes involved in the synthesis of the diterpene *Z*-abienol [13], although demonstration of enzymatic activity of the tomato proteins encoded by these genes is still lacking [11]. The fifth TPS gene in this cluster, *TPS21*, was shown to encode a protein that in vitro uses NNPP to produce a previously unknown diterpene [11] that was subsequently structurally characterized and named lycosantalene [12]. NNPP is the product of the second CPT gene, *CPT2*, that is present in the gene cluster on chromosome 8 (Fig. 1A). Furthermore, by co-expressing *CPT2*, *TPS21*, and *CYP71BN1* in a bacterial system we were able to show that the CYP71BN1 enzyme is able to catalyze two sequential oxidation reactions of lycosanatalene to produce first epoxy-lycosantalene and then lycosantalonol (Fig. 1B, [12]).

To date, however, lycosanatalene and its oxidative derivatives have not been identified in any tomato tissue. Here we show that *TPS21*, *CPT2* and *CYP71BN1* are expressed at their highest levels in leaf petiolules, and that this part of the plant is likely to naturally produce compounds derived from NNPP.

## Materials and Methods

### Chemicals

DMAPP, IPP, and *zz*FPP were obtained from Echelon Biosciences. Radio-labeled ^14^C–IPP, 60.0 mCi/mmol (2.22 GBq/mmol), was obtained from Perkin Elmer. Solid-phase microextraction (SPME) fiber was obtained from Supelco. All other chemicals were obtained from Sigma, Promega, Invitrogen or Qiagen.

### Tissue isolation and preparation of petiolules, petiolules without trichomes, and trichomes from petiolules for RNA extractrion and metabolite analysis

For analysis of terpenoids of whole petiolules, 20–30 mg of petiolules of compound leaves from all developmental stages were grounded in a microtube and 100 μl of hexane were added into the tube and incubated for 10 min. Water was then added, the sample was vortexed and centrifuged, the hexane phase was analyzed by GC-MS. For analysis of terpenoids in trichomes, 20–30 mg of intact petiolules were placed in a microtube and 100 μl of hexane were added into the tube and incubated for 10 min (the “dip” method), and then the hexane was removed and analyzed by GC-MS. To measure terpenoid content in the non-trichome portion of the petiolules, petiolules that were first dipped in hexane (to remove terpenoids from the trichomes) were placed into a fresh microtube, ground, and extracted with 100 μl of hexane, and the hexane extracts were analyzed by GC-MS.

For the qRT-PCR analysis, intact whole petiolules were ground in liquid nitrogen and total RNA was extracted. To obtain petiolules without trichomes and pure trichome preparations, intact petiolules were first placed in a microtube and frozen with liquid nitrogen. Vortexing the tube caused the trichomes to separate from the petiolule tissue. Using a tweezer, the petiolules without trichomes were removed and placed in a new tube, ground, and total RNA was extracted. The remaining trichomes were collected by centrifugation and used for total RNA extraction as well.

### Gene expression analysis of *CPT2*, *TPS21* and *CYP71BN1* by qRT-PCR

After total RNA was isolated with the E.Z.N.A. Plant RNA MiniKit (Omega Bio-tek), it was treated with a DNA-free kit (Ambion) to remove genomic DNA contamination, and used for first-strand cDNA synthesis with a High Capacity cDNA reverse transcription kit and random primers (Applied Biosystems) according to the manufacturer’s protocol. To quantify the mRNA abundance of *CPT2*, *TPS21* and *CYP71BN1* in each tissue in *S*. *lycopersicum*, quantitative RT-PCR was performed as previously described [10, 11]. To compare absolute expression levels among the three genes, the standard curve method was used. Relative expression levels in different tissues were normalized to the expression levels of tomato elongation factor-1a (EF-1 a; GenBank: X14449). All primers used in this study are shown in S1 Table. Three or four biological replicates (as indicated in the legends) were used for each point, and triplicates of each sample were done.

### Transgenic plants

For the *35S-CPT2* construct, the open reading frame of the *CPT2* gene was amplified with KOD polymerase using pGEM-T Easy-*CPT2* plasmid as a template and ligated into pSAT4A vector [14] between SalI/BamHI restriction sites. The region including the double 35S CaMV promoter, enhancer, *CPT2* gene, and the terminator of pSAT4A-*CPT2* was digested with I-SceI restriction enzyme and ligated into the I-SceI site of the binary vector, pPZP-RCS2 [15]. For the *35S-CPT2*-RNA interference (RNAi) construct, a 190 bp fragment of *CPT2* that corresponds to nucleotides 54 to 243 of the gene was amplified by PCR. The fragment was ligated in the sense and antisense orientations into pRNA69 [16] between the XhoI/KpnI and BamHI/XbaI restriction sites, respectively. The hairpin cassette was released by SpeI/SacI digestion and transferred to the pZP212 binary vector [17] between the XbaI/SacI restriction sites. All primers used are shown in S1 Table. The binary vectors were introduced into *S*. *lycopersicum* cultivar MP1 by the University of Nebraska Plant Transformation Facility (http://biotech.unl.edu/plant-transformation). All transgenic plants were grown at the growth room at the same condition as previously described [11] and the first generation of *35S-CPT2* and *35S-CPT2*-RNAi transgenic lines were used for the metabolic analyses. *CPT2* transcript levels of individual *35S-CPT2* and *35S*-*CPT2*-RNAi transgenic plants were analyzed by RT-PCR and qRT-PCR, using total RNA obtained from terminal leaflets and petiolules, respectively, with primers shown in S1 Table.

### Terpenoids analysis by GC-MS

Terpenoids were collected by extraction with hexane from plant tissues or by SPME of ground plant samples placed in a 2 ml glass vial for 15 min at 42°C. To analyze the accumulation of terpenoid glucosides, glucosides were extracted with 80% methanol from ground tissue for 16 hours at 4°C. The extracts were filtered and solvent evaporated without heating in a SpeedVac concentrator (Savant). Compounds were dissolved in 50 mM citrate buffer pH 5.0, and β-glucosidase was added and the sample was incubated for 16 hours at 37°C, or dissolved in either 0.5 N HCl or 0.5 N NaOH and incubated for 1 hour at room temperature, and then neutralized with NaOH or HCl. Nonpolar compounds were extracted with hexane and the samples were analyzed by GC-MS. Samples were injected in split mode (2:1) into a Rxi-5Sil MS column (30-m length, 0.25-μm film thickness, and 0.25-mm ID; Restek) on a GC-2010 Plus coupled to a GCMS-QP2010 SE (Shimadzu) using 70 eV electron ionization. Injector temperature was 240°C, and interface temperature was 280°C. The following GC methods were used: After a 3-min isothermal hold at 150°C, the column temperature was increased by 3°C/min to 240°C. Lycosantalene, nerylnerol, lycosantalonol, and epoxy-lycosantalene obtained by engineering *E*. *coli* cells and verified by NMR [12] were used as standards.

### Terpenoids analysis by HPLC-MS

The 80% methanol extract of ground petiolules was analyzed using high-performance liquid chromatography (HPLC) (LC-20AD pump, CTO-20A column oven, and SIL-5000 autosampler, Shimadzu) using an ODS column (Ascentis Express C_18_, i.d. 2.1 x 100 mm, Supelco) coupled with time-of-flight (TOF)-MS (LCT Premier TOF-MS, Waters) with ES negative ion mode from *m/z* 50 to 1500 using three multiplexed collision-induced dissociation functions (Aperture 1 voltages were 10, 20, and 30 V) with 0.3 s per scan for each function. Metabolites were separated by linear gradient elution (0.3 ml/min) from 40 to 100% solvent B (100% methanol) in solvent A (0.15% formic acid in water) for 14 min. All data were analyzed with MassLynx V4.1 software (Waters).

### Recombinant CPT2 characterization

Recombinant CPT2 protein was generated in *E*. *coli* BL21 (DE3) that harbored the expression vector pEXP5-CT/TOPO (Invitrogen) containing *CPT2* cDNA with a deletion of the 153 nucleotide sequences corresponding to the 51 amino acids of the N-terminal putative transit peptide. *E*. *coli* harboring *CPT2*-pEXP5-CT/TOPO were incubated in Luria-Bertani media at 30°C until they reach OD_600_ = 0.8. The production of the CPT2 protein was then induced by adding 1 mM isopropyl-β-D-1-thiogalactopyranoside as a final concentration and incubating the culture for 18 hours at 16°C. The recombinant CPT2 protein was purified by HIS-Select HF Nickel Affinity Gel (Sigma) according to the manufacturer’s protocol. Kinetic analysis was performed using ^14^C-IPP as a substrate with 3.0 μg of purified recombinant enzyme in each reaction. The pH preference of the enzyme was determined by enzymatic assays with 0.5 μg purified enzyme using different pHs in buffers including 100 mM KCl, 7.5 mM MgCl_2_, 5% (v/v) glycerol, and 5 mM DTT. The reaction mixture, including 40 μM DMAPP and 40 μM ^14^C-IPP with a total volume of 50 μl was incubated at 30°C for 15 min. The reaction was stopped and the phosphate groups were removed from the enzymatic products by adding 1 volume of 1 N HCl and incubating the samples at 37°C for 30 min. The hydrolyzed radio-labeled products were extracted with 150 μl of ethyl acetate, and the radio-labeled products were quantified in 100 μl of the extract by scintillation counting. Estimates of the *K*_*m*_ for *zz*FPP and IPP were performed over a range of substrate concentrations using a fixed concentration of the co-substrate as follow: 5–80 μM ^14^C-IPP at 150 μM *zz*FPP and 10–80 μM *zz*FPP at 50 μM ^14^C-IPP. All other conditions were same as previously described [18].

## Results

### Optimal transcript levels of *CPT2*, *TPS21* and *CYP71BN1* are observed in leaf petiolules

**Fig 2:**
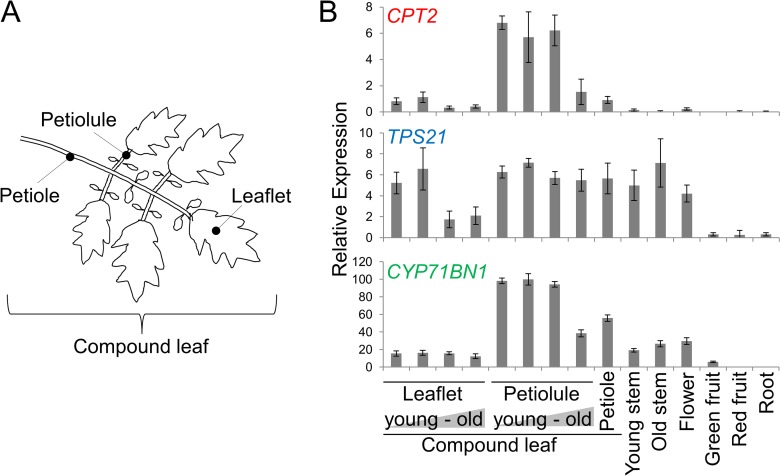
qRT-PCR analyses of *CPT2*, *TPS21* and *CYP71BN1* transcripts in different tissues of *S*. *lycopersicum*. Total RNA was isolated from various tomato tissues. Leaflets and petiolules were prepared from four different developmental compound leaf stages. Error bars represent SE. Values are from three biological and three technical replicates.

Because we previously looked at organ-specific expression of *CPT2*, *TPS21* and *CYP71BN1* by measuring transcripts extracted from whole organs, it was possible that the overall low-level expression observed in these genes was masking high-level expression in small parts of a given organ or at different stages of development. We therefore examined transcript levels of these genes in sub-sections of the compound tomato leaf and in young vs. old organs of the plant (Fig. 2A). There was some variation in the patterns of transcript levels among the three genes. *TPS21* transcripts were present in various parts in relatively similar levels, with the exception of fruits and roots, where the levels were quite low (Fig. 2B). *CYP71BN1* transcript levels were highest in petiolules, the stem-like structure connecting the leaflet to the main petiole, and particularly in young petiolules, but also present in petioles, stems and flowers (Fig. 2B). However, *CPT2* transcripts were present in young petiolules at levels that were at least 4-fold higher than in any other tissue examined (Fig. 2B). The transcripts of all three genes were at their highest levels in young petiolules, compare with other tissues examined here. However, the relative transcript levels of the three genes were different. While the transcript levels of *CPT2* and *TPS21* were almost same, the transcript levels of *CYP71BN1* were approximately 10-fold higher than the levels of the other two genes (Fig. 2B).

**Fig 3:**
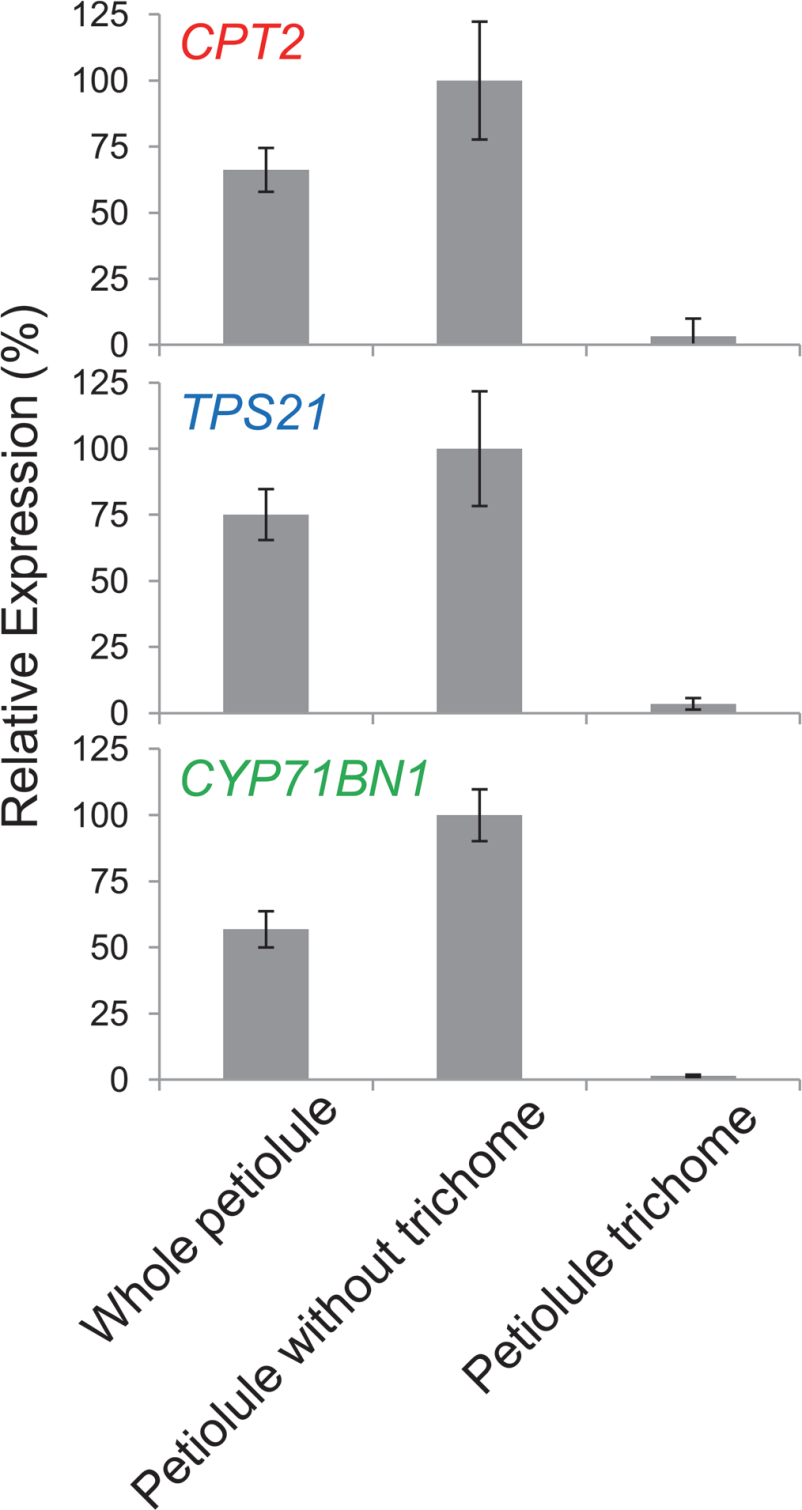
qRT-PCR analysis of *CPT2*, *TPS21*, *CYP71BN1* transcripts in petiolules. RNA was isolated from whole petiolule, petiolules from which trichomes have been removed, and from the trichomes. Error bars represent SE. Values are from four biological replicates with three technical replicates of each.

We also investigated whether *CPT2*, *TPS21* and *CYP71BN1* gene transcripts are present specifically in trichome. Relative levels of *CPT2*, *TPS21* and *CYP71BN1* transcript in petiolules from which trichomes have been removed (see M&M) were 1.5-, 1.3- and 1.7-fold, respectively, higher compare with their levels in whole petiolules (including trichomes), indicating that transcripts of these three genes are present mostly in non-trichome cells in this organ (Fig. 3). Consistent with this conclusion, the relative transcript levels of the three genes in the trichomes of petiolules were respectively 22-, 21- and 40-fold lower compare with their levels in whole petiolules (Fig. 3).

### Over-expressesing *CPT2* in petiolules results in detectable levels of lycosantalene and lycosantalonol

**Fig 4:**
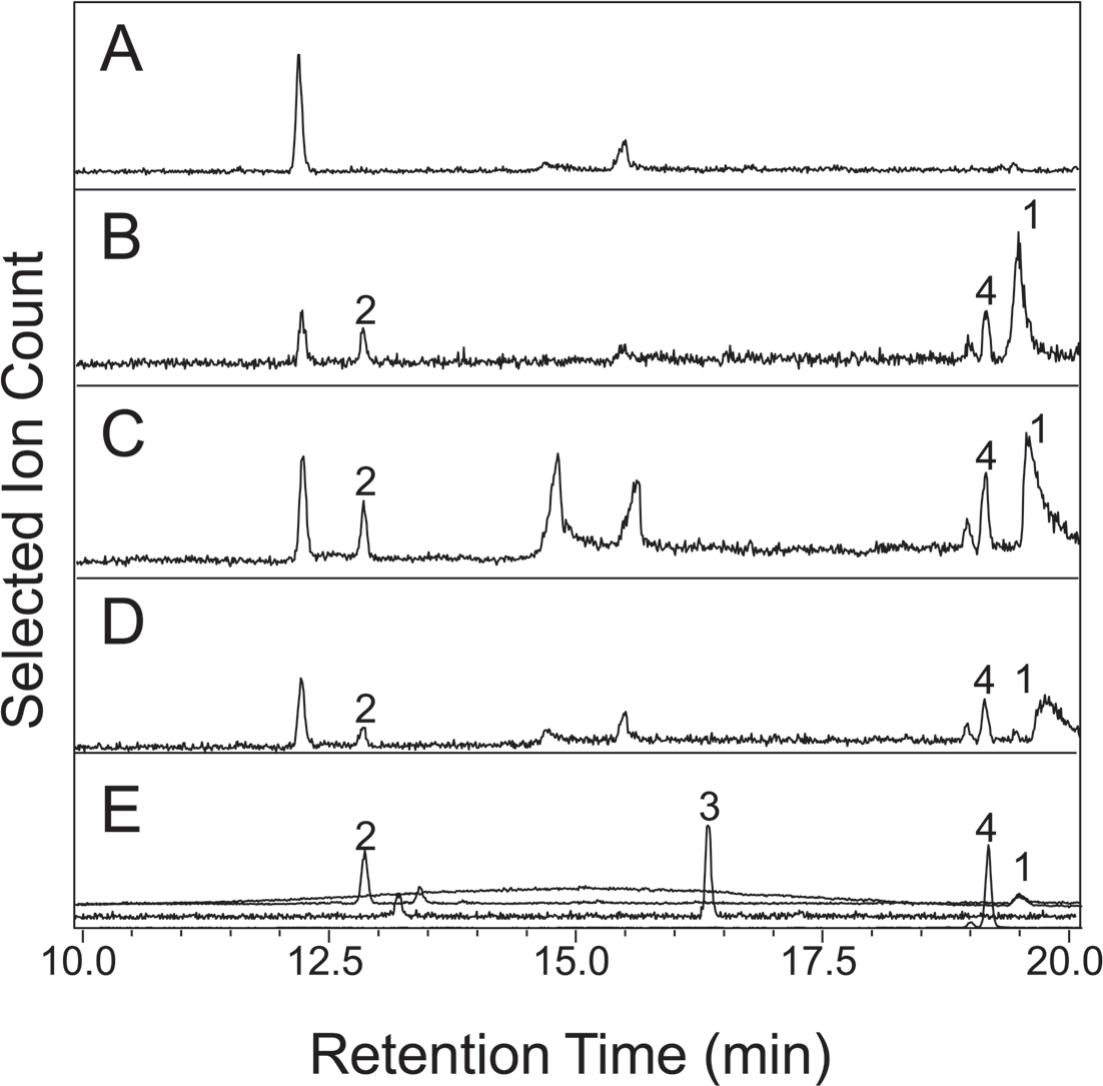
GC-MS analysis of diterpenes from hexane extracts of whole petiolules. (**A**) Non-transformed *S*. *lycopersicum*, (**B**–**D**) three individual plants of *S*. *lycopersicum* transformed with the *35S-*CPT2 gene construct. Chromatograms of the selected ion of *m/z* 109 are shown here. (**E**) Four combined chromatographs of authentic standards. 1, nerylnerol; 2, lycosantalene: 3, epoxy-lycosantalene; 4, lycosantalonol. Mass Spectra for all peaks is shown in S2 Fig. Whole petiolules were ground and extracted with hexane as described in Materials and Methods.

Since the maximal levels of transcripts for all three genes *CPT2*, *TPS21* and *CYP71BN1* occur in young petiolule, we searched for lycosantalonol or related compounds with the basic lycosantalene skeleton in this organ in wild-type plants. Petiolules were placed in a glass vial and ground with a glass stick. Volatiles were collected by SPME at 42°C for 15 min and analyzed by GC-MS. For non-volatile terpenoids analysis, compounds were extracted from the ground petiolules with hexane as a solvent and the extracts were analyzed by GC-MS. To investigate the accumulation of diterpenoid glucosides or diterpenoids that were modified by acylation, compounds were extracted from the ground petiolules with 80% methanol, and treated with either acid or base (final concentration 0.5 N HCl or 0.5 N NaOH, respectively), or dried first, then resuspended in buffer and incubated with almond recombinant β-glucosidase. After these treatments, compounds were extracted with hexane and analyzed by GC-MS. No peaks with the retention times of lycosantalene, epoxy-lycosantalene, or lycosantalonol were detected in the chromatograms of samples obtained by any of the methods described above (one such result is shown in Fig. 4A). Furthermore, we did not detect any other peaks in which at least two of the ions of *m/z* 69, 81, 93, 95, 107, 109 and 121, which are prominent in the MS of lycosantalene and/or its derivatives, constituted a significant part of their mass spectra.

Since it was possible that such compounds were produced in the wild-type petiolules at such low levels as to be below the detection threshold of our methods, and that increasing the rate of synthesis of NNPP would lead to higher levels of synthesis of lycosantalene and its derivatives, we constructed transgenic tomato plants containing the *CPT2* gene under the control of the strong, non-specific 35S promoter. *CPT2* expression level of individual transgenic plants was determined by RT-PCR (S1 Fig.). SPME analysis of transgenic leaflets of plants over-expressing *CPT2* detected lycosantalene in eight of the 13 individual transgenic plants tested. Three individual plants which had the highest lycosantalene levels were selected for further analyses. Petiolules were collected from these three individual transgenic tomato lines and diterpenoids were extracted with hexane and analyzed by GC-MS. Nerylnerol, lycosantalene and lycosantalonol were all detected in all three individual plants (Fig. 4B-D and S2 Fig.). To investigate the accumulation of diterpenoid glucosides or diterpenoids that were modified by acylation, compounds were extracted from ground petiolules and analyzed in the same way as described above for wild-type petiolules. However, no additional lycosantalene derivatives were found in the transgenic petiolules in any of these three lines.

### Petiolules of *CPT2*-overexpressing lines produce neryneryl diphosphate-derived diterpenes in non-trichomes cells

Tomato trichomes have high levels of the monoterpene β-phellandrene and the sesquiterpene β-caryophyllene, which are present at much lower levels in other aerial cells [10]. We analyzed hexane extracts of ground whole petiolules of transgenic plants overexpressing *CPT2* ground, hexane extracts of ground petiolules from which the terpenes in the trichomes had first been removed by dipping the sample in hexane, as well as terpenoid content of the hexane solution in which the petiolules were dipped in. We measured lycosantalene rather than lycosanatalonol since the former has a lower detection threshold. This analysis showed that petiolules without trichomes had higher ratios of lycosantalene to β-phellandrene and to β-caryophyllene than petiolules with trichomes (Table 1), indicating that a higher proportion of lycosantalene than β-phellandrene and β-caryophyllene is present in the non-trichome petiolule tissue. The observation that the ratios of lycosantalene to β-phellandrene and to β-caryophyllene were lower in trichomes than in whole petiolules (Table 1) is also consistent with this conclusion.

**Table 1:** Ratios of lycosantalene levels to β-phellandrene and β-caryophyllene levels in petiolule with and without trichomes, and in trichomes of transgenic *S*. *lycopersicum* plants overexpressing *CPT2*.

Tissue	Lycosantalene / β-phellandrene (%)	Lycosantalene / β-caryophyllene (%)
Petiolules without trichomes	6.1 ± 1.1	61.2 ± 8.6
Petiolules with trichomes	4.1 ± 1.1	37.1 ± 9.0
Petiolule trichomes	1.1 ± 0.2	11.4 ± 2.1

Terpenes were extracted with hexane and analyzed by GC-MA as described in Materials and Methods.

### CPT2 shows high affinity for IPP

We showed previously that the preferred initial acceptor substrate of CPT2 is DMAPP [10]. Here we measured the *K*_*m*_ and *k*_*cat*_ values of CPT2 for the condensation of *zz*FPP and IPP, the last step in the three-step reaction of synthesizing NNPP from DMAPP and 3 molecules of IPP. CPT2 showed optimal activity at pH 8.0–8.5, similar to what has been observed with other CPT enzymes (S3 Fig.). The *K*_*m*_ value for IPP was determined to be 3.8 ± 0.3 μM and for *zz*FPP 22.9 ± 1.1 μM, values that are lower than those of SlCPT1 (NDPS1) for its substrates (*K*_*m*_ value for IPP: 152 μM, DMAPP: 177 μM, [8]) and ShCPT1 (zFPS, *K*_*m*_ for IPP: 16, 36 μM, DMAPP: 35, 210 μM, [9]) (Table 2). The measured *k*_*cat*_ value with CPT was similar with both IPP and *zz*FPP substrates, at 2.6 x 10^−3^ (s^−1^).

**Table 2:** NNPS activity of CPT2.

Substrate	*K*_*m*_ (μM)	*k*_*cat*_ (s^−1^)	*k*_*cat*_ /*Km*, s^−1^ mM^−1^
IPP	3.8 ± 0.3	(2.6 ± 0.2) x 10^−3^	0.68 ± 0.04
*zz*FPP	22.9 ± 1.1	(2.6 ± 0.1) x 10^−3^	0.11 ± 0.01

### No obvious morphological phenotypes are observed in *35S-CPT2* or *35S-CPT2-RNAi* tomato lines

Although we were able to detect lycosantalene and lycosantalonol in transgenic tomato plants overexpressing *CPT2* under the control of the 35S promoter, the gross morphology of the plants was similar to that of wild-type plants. We also examined the transgenic tomato plants expressing a *35S-CPT2* RNAi construct in which *CPT2* gene expression was reduced by up to 80% (S4 Fig.), and these plants grew normally and showed no morphological differences from wild-type tomato plants at all developmental stages.

### No differences in metabolic compounds are observed between both *CPT2* over-expressing and RNAi lines and wild-type plants

The volatile and non-volatile hydrophobic compound analyses of petiolules from three individual transgenic plants in which *CPT2* transcript levels were reduced by 80% were performed by GC-MS, and no differences were observed compared with wild-type petiolules.

Next, a detailed hydrophilic metabolic compounds analysis was performed by HPLC-MS. The hydrophilic compounds were extracted with 80% methanol from ground petiolules from each of five individual transgenic plants as well as from control wild-type plants. All 2,370 peaks observed on chromatograms of the five individual *CPT2* over-expression lines (#03, 07, 19, 24, and 26) and/or the five *CPT2* RNAi lines (#01, 04, 05, 12, and 18) were compared to the peaks of the wild-type plants by a global comparison software (MarkerLynx XS, MassLynx, Waters). However, no significant increases or decreases in levels of the peaks among these three groups were observed.

### CYP71BN1 is most similar to germacrene A oxidases

**Fig 5:**
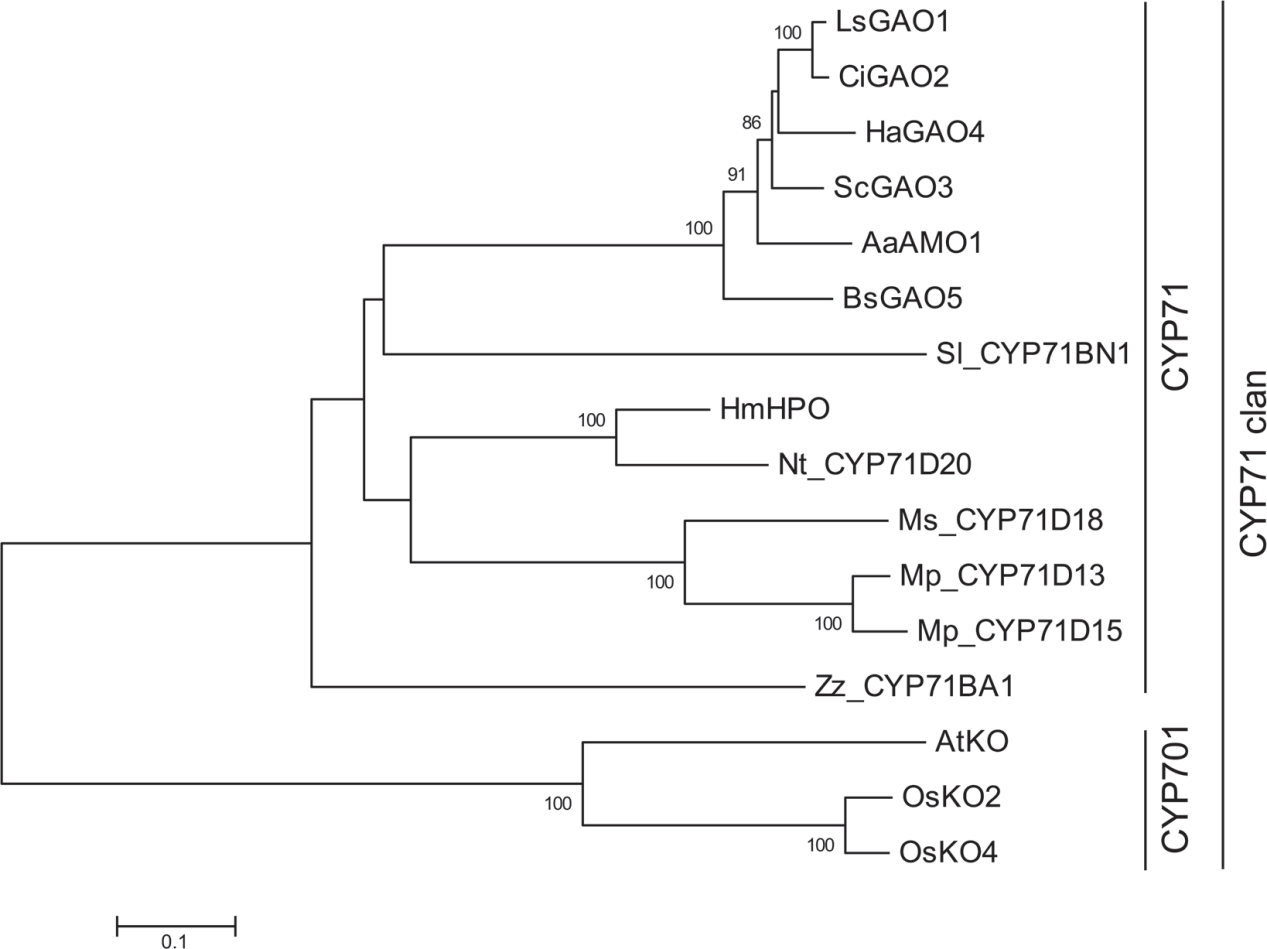
Phylogenetic tree of tomato CYP71BN1 and other functionally characterized terpene-modifying P450s. Neighbor-joining phylogenetic tree analysis using amino acid sequences was performed by MEGA 5 [40]. Bootstrap values were performed with 1000 replications (values shown next to branches). LsGAO1, *Lactuca sativa* germacrene A oxidase (GAO) 1 (ADF32078.1); CiGAO2, *Cichorium intybus* (ADF43080.1); HaGAO4, *Helianthus annuus* (ADF43082.1); ScGAO3, *Saussurea costus* (ADF43081.1); AaAMO1, *Artemisia annua* amorpha-4, 11-diene monooxygenase (Q1PS23.1); BsGAO5, *Barnadesia spinosa* (ADF43083.1); HmHPO, *Hyoscyamus muticus* premnaspirodiene oxygenase (HPO) (A6YIH8.1); Nt-CYP71D20, *Nicotiana tabacum* 5-epiaristolochene dihydroxylase (Q94FM7.2); Ms-CYP71D18, *Mentha spicata* (-)-(4*S*)-limonene-6-hydroxylase (Q9XHE8.1); Mp-CYP71D13, *Mentha x piperita* (-)-(4*S*)-limonene-3-hydroxylase (Q9XHE7.1); Mp-CYP71D15, *Mentha x piperita* (-)-(4*S*)-limonene-3-hydroxylase (Q9XHE6.1); Zz-CYP71BA1, *Zingiber zerumbet* α-humulene oxidase (E3W9C4.1); AtKO, *Arabidopsis thaliana ent*-kaurene oxidase (KO) (NM_122491); OsKO2, *Oryza sativa* (BAF19823); OsKO4, *Oryza sativa* (BAF19823).

Cytochrome P450 oxidoreductases belonging to the CYP71 clan are known to be involved in the biosynthesis of specialized metabolites such as isoprenoids, alkaloids, flavonoids and cyanogenic glucosides. Most mono-, sesqui- and diterpene-modifying P450s belong to this CYP71 clan [19]. Phylogenetic tree analysis showed that tomato CYP71BN1 was most similar to sesquiterpene oxidases, germacrene A oxidases (GAOs) from both Asteraecae and Barnadesioideae and amorpha-4, 11-diene oxidase (AMO) from Asteraecae (Fig. 5). GAOs and AMO each catalyze three consecutive oxidations of sesquiterpenes, germacrene A to yield germacrene A acid, in the case of GAO, and amorpha-4,11-dine to yield amorpha-4, 11-dine acid in the case of AMO [20–22]. Tomato CYP71BN1 catalyzes the two sequential oxidations of the diterpene, lycosantalene to yield lycosantalonol (Fig. 1).

## Discussions

### CPT2, TPS21 and CYP71BN1 catalyze the steps in the biosynthesis of lycosantalonol in petiolules

Gene expression analyses revealed that *CPT2*, *TPS21* and *CYP71BN1* co-express in the petiolule, but not in the trichomes of petiolule. While we did not detect any lycosantalene, lycosantalonol, or their derivatives in the petiolules or any other part of the wild-type plants, including leaflets, petioles and stems where these genes are also appreciably expressed although at lower levels than in petiolules (Fig. 2), we did detect lycosantalene and lycosantalonol in the petiolules of transgenic tomato expressing *CPT2* under the control of the CaMV 35S promoter. This observation suggests that the amount of NNPP in this tissue limits the amount of the final product of the pathway. Since *CPT2* as well as *TPS21* and *CYP71BN1* are expressed at some level in petiolules, and more so than in any other tissues examined, it is likely that lycosantalene and lycosantalonol are also synthesized in petiolules of non-transgenic tomato plants, and perhaps in other tissues as well, but at levels too low to be detected with our analytical methods. A corollary hypothesis is that lycosantalene and lycosantalonol are intermediates of unknown final metabolite(s), and they only accumulate when the flux is increased artificially, as by overexpressing *CPT2*. Other groups have reported the accumulation of intermediates in transgenic plants but not in wild-type plants, in some cases without yet identifying the final products (for instance β-amyrin in rice [23]).

We had previously reported that the CPT2 protein is localized to the plastids [10]. While the entire pathway to lycosantalonol may not necessarily need to occur in the same compartment—examples of pathways for which enzymes are found in different compartments and where intermediates move from one compartment to another abound (for instance, sesqui- and diterpeniod [24], anthocyanins [25] and benzoxazinoid [26])—we note that the sequences of both *TPS21* and *CYP71BN1* are predicted by WoLFPSORT (http://www.wolfpsort.org/) to encode an N-terminal transit peptide that would target the proteins to the plastid. Furthermore, the sequence of the transit peptide of TPS21 is similar to that of ShTPS45 (zFPS), a protein that was experimentally shown to localize to plastids [9].

The possible role of lycosantalonol or its derivatives in the petiolules is not yet known. While the expression of genes encoding some leaf terpenes has been shown to be induced by herbivory or fungal attack, our attempts to find conditions under which *CPT2* or *TPS21* are induced, for example by using alamethicin [27] have failed to identify such conditions. Since wild-type petiolules make at most low levels of lycosantalonol, this compounds or its derivatives are unlikely to be involved in direct defense.

### Evolution of *cis*-prenyltransferases that produce short-chain prenyl diphosphates and terpene synthases that use all-*cis*-prenyldiphohsphates

The genomes of most plant species examined appear to have a small (<10 members) family of CPT genes. However, these genes are believed to be involved in the biosynthesis of polyprenols (>C_35_) [28]. Only in the genus *Solanum* (Solanaceae) have CPT genes been found that are involved in the synthesis of precursors of C_10_-C_20_ terpenes [8, 9, 11, 12, 18, 29], although analysis of the recently released genome sequence of *Nicotiana tomentosiformis* and *N*. *sylvestiris* reveal close homologs of *SlCPT6*, which encodes a *z*FPS, but no close homologs of *SlCPT2* (Fig. 5). The limited distribution of CPTs for the synthesis of C_10_-C_20_ prenyldiphosphates to Solanaceae, and in particular of CPT2 to the *Solanum* genus, suggests that such enzymes are evolutionary novelties and that the ability to synthesize lycosantalene and lycosantalonol is probably confined to *Solanum* or at most to Solanaceae.

In a previous study, we suggested that *TPS21* was created by a duplication of an ancestral gene likely to encode a diterpene, and whose other progeny is *TPS18*. A second duplication, in the *TPS21* gene lineage, gave rise to an ancestral *TPS19*/*TPS20* lineage (which then duplicated to *TPS19* and *TPS20*) [11]. Based on the position of *TPS21* in the phylogenetic tree and its activity with NNPP, we further hypothesized that the ability to use *cis*-prenyl diphosphates arose first in the ancestral *TPS21* lineage. Although CPT2 had been shown to be able to use NPP and *zz*FPP as substrates, its catalytic efficiency with these substrates is lower than with DMAPP as substrate [10]. These data are consistent with CPT2 having a similar catalytic activity to that of the ancestral *cis*-prenyltransferase in the cluster, since it is not dependent on other *cis*-prenyl diphosphates for its substrate and therefore did not have to evolve only after the others had. We also note that the *K*_*m*_ value of CPT2 for IPP is lower than of other CPTs in the terpenoid gene cluster on chromosome 8.

### Origin of CYP71BN1

Cytochrome P450 oxidoreductases are known to catalyze reactions including hydroxylation, peroxidation and epoxidation. These enzymes are categorized into clades based on their amino acid sequences [30]. Their catalytic mechanism has been well studied and individual family members have been shown to have slightly different helical structures that might be associated with different substrate binding. CYP71BN1 catalyzes the two-step oxidation of lycosantalene. The CYP71 enzymes most similar in primary sequence to CYP71BN1 are GAO and AMO, each of which catalyzes three sequential oxidations of a sesquiterpene to produce a sesquiterpene acid. CYP71s, including GAO and AMO, appear to have evolved from the CYP701 family, whose members include the enzyme that catalyzes the three-step oxidation of the diterpene *ent*-kaurene to form *ent*-kaurenoic acid, a precursor of the plant gibberellin hormones (Fig. 5, [17]). This *ent*-kaurene oxidase (KO), which is found in all land plants, is the only primary metabolism enzyme in the CYP71 clan. Furthermore, both enzymatic products of GAO and AMO, germacrene A acid and amorpha-4,11-dine acid, respectively, are intermediates of the final metabolic products, which are sesquiterpene lactones (costunolide and artemisinin, respectively). These examples, and the observation that no lycosantalonol could be detected in wild-type tomato plants, suggest that lycosantalonol only serves as an intermediate and is further metabolized by other enzymes in tomato, although we have not yet identified such enzymes.

### Evolution of the functional terpenoid gene cluster on chromosome 8 of tomato

Recently, multiple examples of “clusters” of genes that are involved in specifying enzymes for the same pathway of specialized metabolism have been identified in plants [31–38]. We have recently reported that the three genes *CPT2*, *TPS21* and *CYP71BN1*, involved in the biosynthesis of the novel diterpenoid lycosantalonol, are located next to each other in a narrow region of 20 kb on chromosome 8 in *S*. *lycopersicum* [11]. *CPT1* (*NDPS1*), *TPS19*, and *TPS20* (*PHS*), which are responsible for the biosynthesis of the trichome-specific monoterpene β-phellandrene, are located next to these three genes, as are two other TPS genes, *TPS18* and *TPS41*, that encode proteins whose biochemical activities have not yet been determined. A very similar gene cluster with the same gene order has also been found on chromosome 8 of the wild tomato species *S*. *pimpinellifolium* [11]. However, in other species in *Solanum* that have been examined—*S*. *pennellii*, *S*. *habrohaites*, and *S*. *tuberosum*—*CPT2*, *TPS21*, and/or *CYP71BN1* contain small or large deletions that clearly render them inactive. Inactivation by deletions is often observed in genes that once encoded enzymes of specialized metabolism [39]. Since plant specialized metabolites co-evolve with pathogens and herbivores or as adaptations to environmental stress, it is not surprising that pathways of specialized metabolites are inherently unstable and exhibit fast rates of evolution.

## Supporting Information

S1 Fig RT-PCR analysis to identify 35S-*CPT2* transgenic plants with higher levels of *SlCPT2* transcripts compared to non-transgenic plants. Transgenic plants lines CPT2oe-03, 19 and 22 were further analyzed for their diterpenoid content by GC-MS. Lines CPT2oe-03, 07, 19, 24 and 26 were used for metabolic analysis by HPLC-MS.
(TIF)

S2 Fig Mass spectra of compounds corresponding to peaks in Fig. 4. (TIF)

S3 Fig Determination of pH optimum of activity of purified recombinant CPT2. Buffers used were 50 mM citrate buffer (pH 3.0–5.5), 50 mM phosphate buffer (pH 6.0–8.0) and 50 mM Tris-HCl buffer (pH 8.0–9.5).
(TIF)

S4 Fig Petiolule *CPT2* transcript levels in 16 transgenic plants expressing the 35S-CPT2-RNAi construct compared to wild-type *CPT2* transcript levels. Total RNA was extracted from petiolule tissue. Lines CPT2i-01, 04, 05, 12 and 18, which had the lowest *CPT2* transcript levels, were used for metabolic analysis.
(TIF)

S5 Fig Neighbor-joining phylogenetic tree analysis of *Solanum* and *Nicotiana* CPTs. The sizes of the polyisoprenoids of characterized enzymes are shown inside brackets. Nt, *Nicotiana tomentosiformis*; Ns, *Nicotiana sylvestris*.
(TIF)

S1 Table Synthetic oligonucleotides used in this study. (PDF)
