# Supplementary figures and images for: Biosynthesis of the Diterpenoid Lycosantalonol via Nerylneryl Diphosphate in Solanum lycopersicum
Source: PLoS One. 2015 Mar 18;10(3):e0119302. doi: 10.1371/journal.pone.0119302 (PMC4364678; doi:10.1371/journal.pone.0119302)

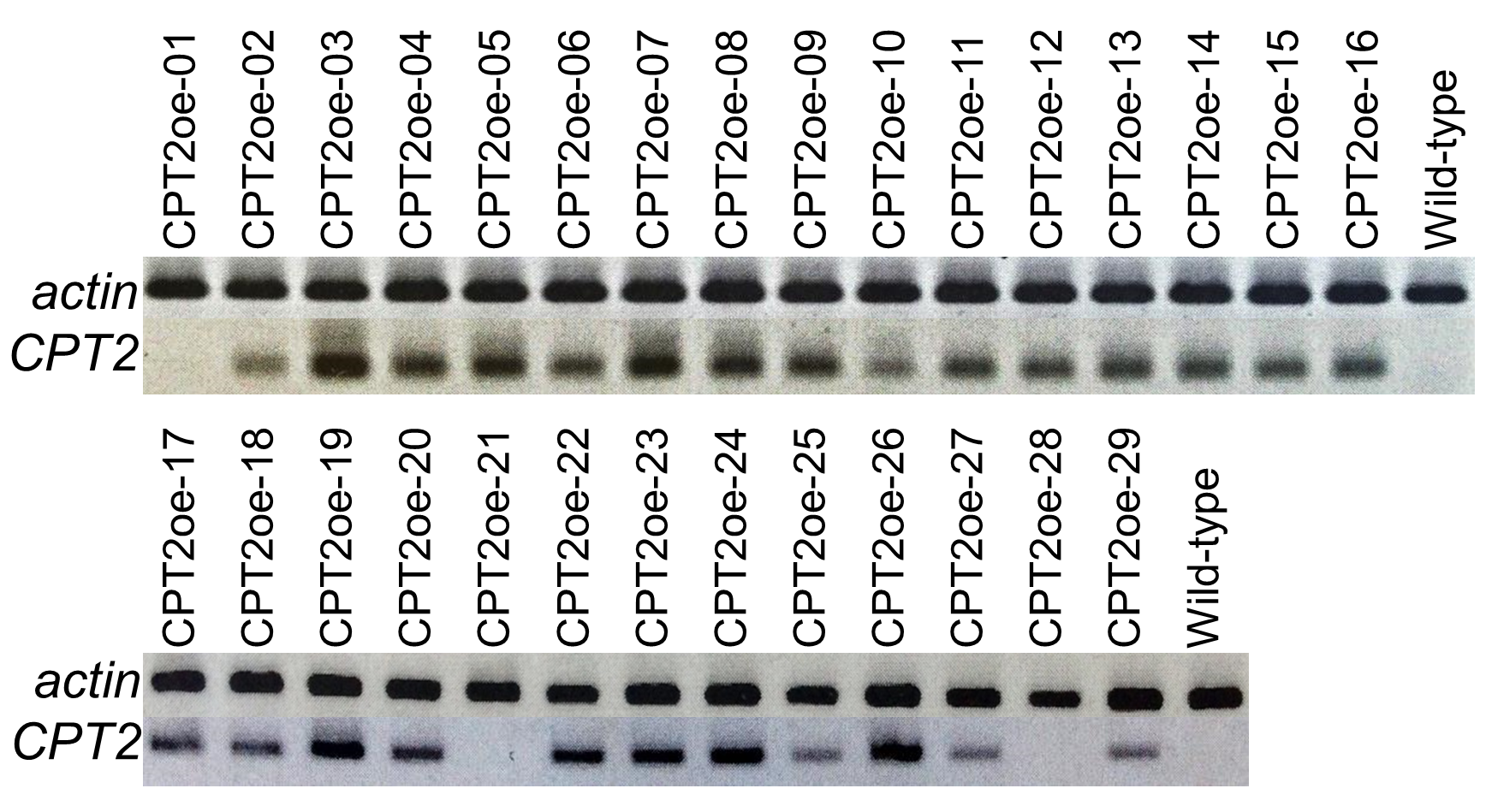

Supplement: S1 Fig — Transgenic plants lines CPT2oe-03, 19 and 22 were further analyzed for their diterpenoid content by GC-MS. Lines CPT2oe-03, 07, 19, 24 and 26 were used for metabolic analysis by HPLC-MS. (TIF) [file pone.0119302.s001.tif]

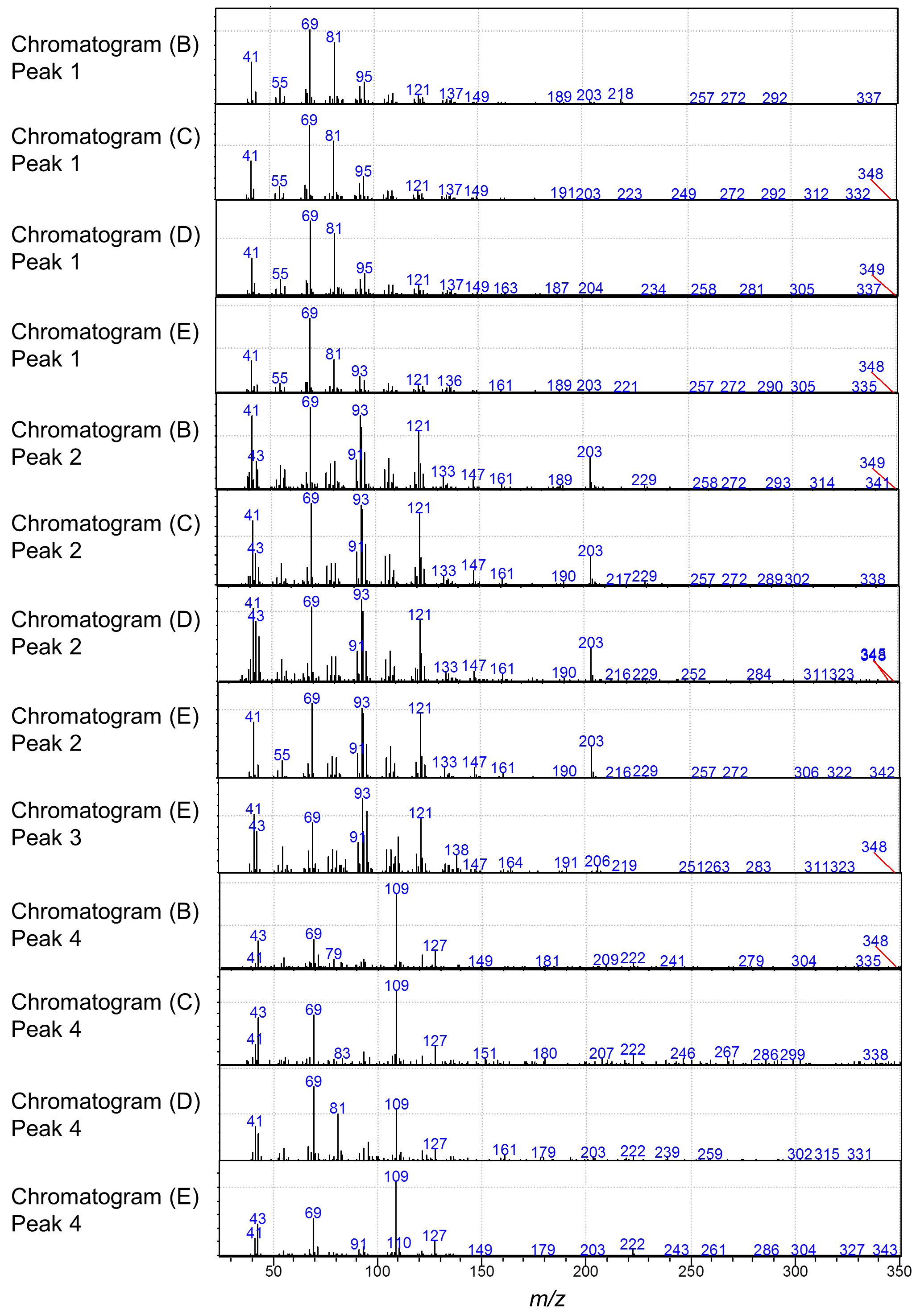

Supplement: S2 Fig — (TIF) [file pone.0119302.s002.tif]

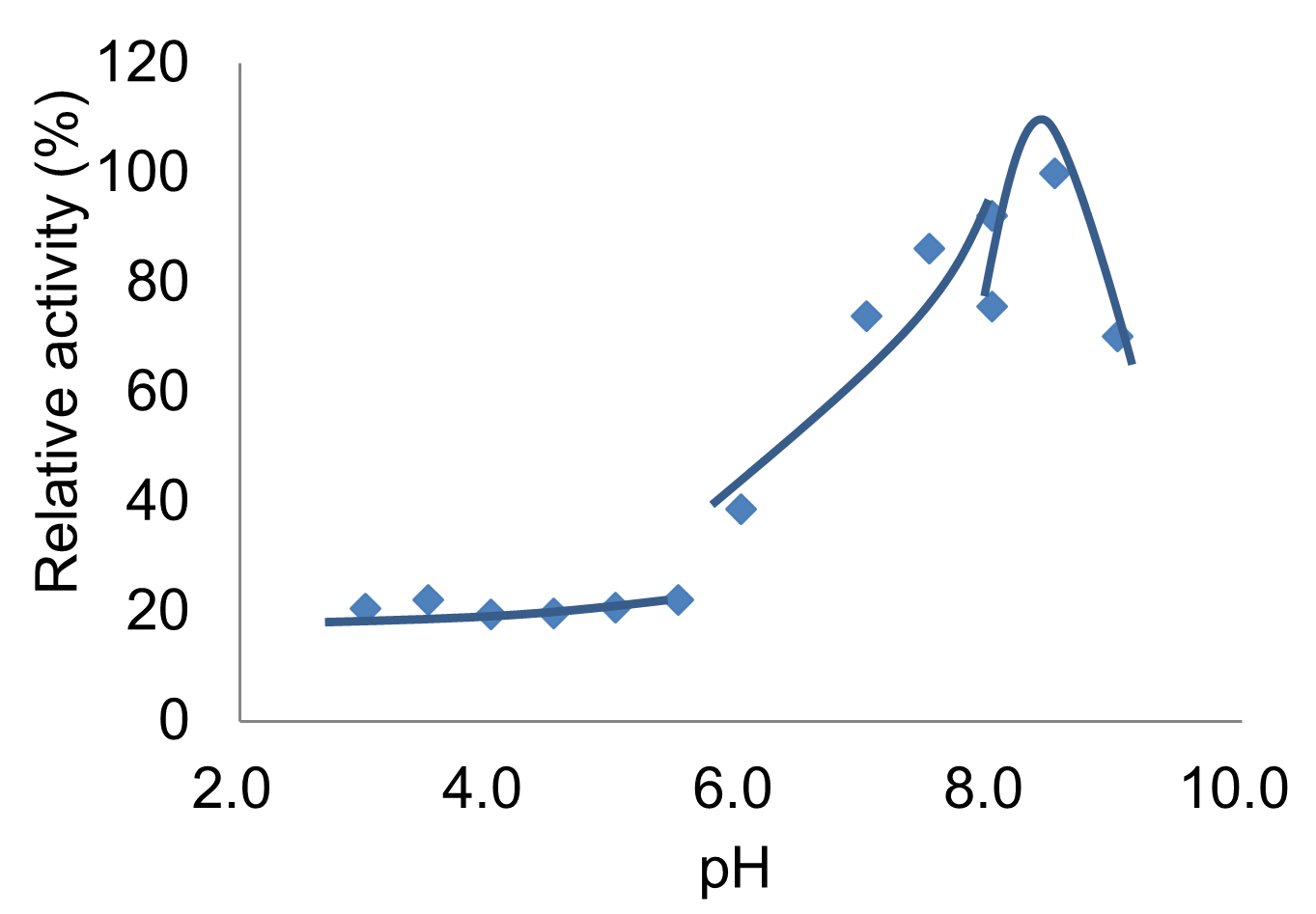

Supplement: S3 Fig — Buffers used were 50 mM citrate buffer (pH 3.0–5.5), 50 mM phosphate buffer (pH 6.0–8.0) and 50 mM Tris-HCl buffer (pH 8.0–9.5). (TIF) [file pone.0119302.s003.tif]

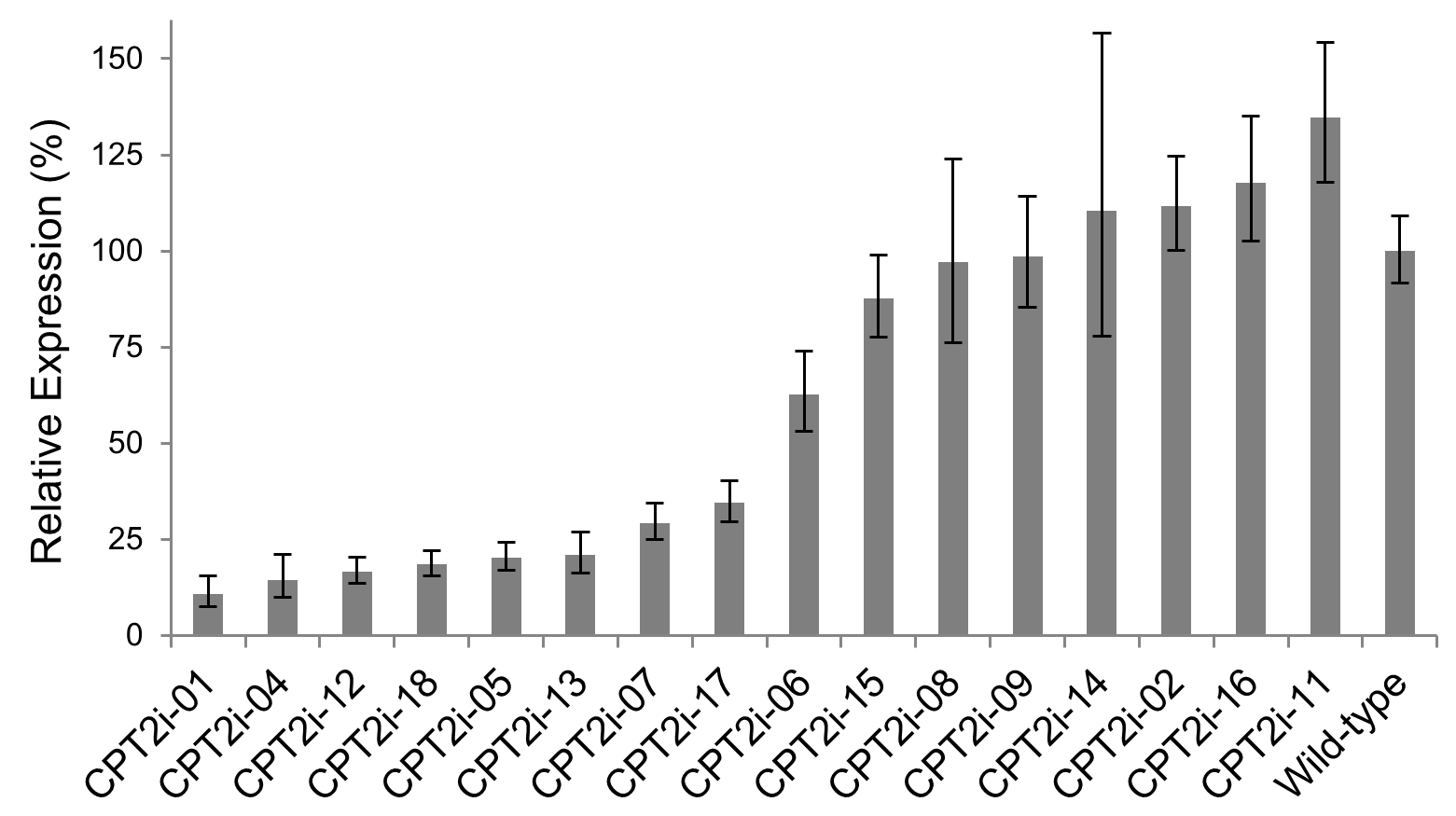

Supplement: S4 Fig — Total RNA was extracted from petiolule tissue. Lines CPT2i-01, 04, 05, 12 and 18, which had the lowest CPT2 transcript levels, were used for metabolic analysis. (TIF) [file pone.0119302.s004.tif]

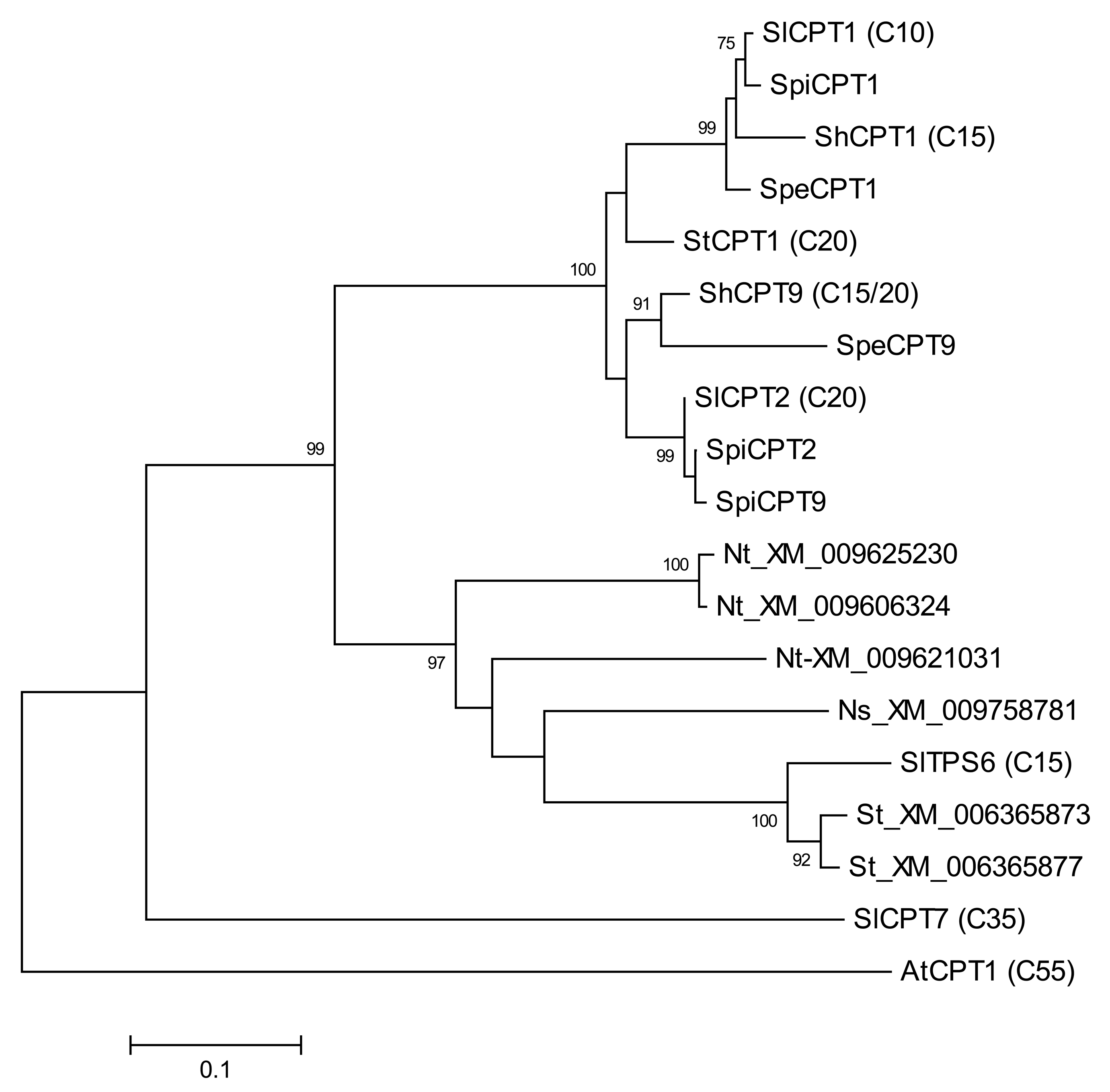

Supplement: S5 Fig — The sizes of the polyisoprenoids of characterized enzymes are shown inside brackets. Nt, Nicotiana tomentosiformis; Ns, Nicotiana sylvestris. (TIF) [file pone.0119302.s005.tif]
