# Supplementary material for: Biosynthesis of the Diterpenoid Lycosantalonol via Nerylneryl Diphosphate in Solanum lycopersicum
Source: PLoS One. 2015 Mar 18;10(3):e0119302. doi: 10.1371/journal.pone.0119302 (PMC4364678; doi:10.1371/journal.pone.0119302)
Supplement: S1 Table — (PDF) [file pone.0119302.s006.pdf]

S1 Table Synthetic oligonucleotides used in this study.

Primer description	Sequence (5' - 3')
For qRT-PCR	
CPT2-For	GGATCTTCAATGCTGGAAATCATCATCACC
CPT2-Rev	AAGCTCTTCATGAAGCTCGAGATCATC
TPS21-For	AACTCAAGAGGTGGTTTCAAGATTG
TPS21-Rev	CTCAGGTTCAGACACAACAGCTATG
CYP71BN1-For	TTCACGAACACGAAGAAAGTATTAG
CYP71BN1-Rev	TCCACCGATGAACATATCCAAG
EF-1a-For	GCTGCTGTAACAAGATGGATGC
EF-1a-Rev	CCCTTGTACCAGTCGAGGTTG
For transgenic tomato	
CPT2-35S-For	GATTGTCGACATGAACTCTTCAATAGTGTCTC
CPT2-35S-Rev	CTAAGGATCCTCAATATGTGTGTCCACCAAAAC
CPT2-RNAi-XhoI-54	AGAGCTCGAGGGATCTTCAATGCTGGAAATC
CPT2-RNAi-KpnI-243	CATGGGTACCGAGATCATCATTATCATCATTATC
CPT2-RNAi-BamHI-243	CATGGGATCCGAGATCATCATTATCATCATTATC
CPT2-RNAi-XbaI-54	AGAGTCTAGAGGATCTTCAATGCTGGAAATC
For RT-PCR of CPT2 over-expression line	
CPT2-screening-F	TAATGATGATCTCGAGCTTC
CPT2-screening-R	GTTGGATTTACAACCAATAAC
Actin-screening-F	TGCCATGTATGTTGCCATCCAGGC
Actin-screening-R	AGCTCTTCTCAACAGATGAGCTGG

The restriction sites are underlined.
